# Characteristics of martensitic and strain-glass transitions of the Fe-substituted TiNi shape memory alloys probed by transport and thermal measurements

**DOI:** 10.1038/s41598-017-16574-0

**Published:** 2017-11-27

**Authors:** Balakrishnan Ramachandran, Pei-Chi Chang, Yung-Kang Kuo, Chen Chien, Shyi-Kaan Wu

**Affiliations:** 1 Department of Physics, National Dong Hwa University, Hualien, 97401 Taiwan; 2 Department of Materials Science and Engineering, National Taiwan University, Taipei, 10617 Taiwan

## Abstract

The electrical resistivity, Seebeck coefficient, thermal conductivity, and specific heat of Ti_50_Ni_50-*x*_Fe_*x*_ (*x* = 2.0–10.0 at.%) shape memory alloys (SMAs) were measured to investigate the influence of point defects (Fe) on the martensitic transformation characteristics. Our results show that the Ti_50_Ni_48_Fe_2_ and Ti_50_Ni_47_Fe_3_ SMAs have a two-step martensitic transformation (B2 → R and R → B19′), while the Ti_50_Ni_46_Fe_4_, Ti_50_Ni_44.5_Fe_5.5_, and Ti_50_Ni_44_Fe_6_ SMAs display a one-step martensitic transition (B2 → R). However, the compounds Ti_50_Ni_42_Fe_8_ and Ti_50_Ni_40_Fe_10_ show strain glass features (frozen strain-ordered state). Importantly, the induced point defects significantly alter the martensitic transformation characteristics, namely transition temperature and width of thermal hysteresis during the transition. This can be explained by the stabilization of austenite B2 phase upon Fe substitution, which ultimately leads to the decrease in enthalpy that associated to the martensitic transition. To determine the boundary composition that separates the R-phase and strain glass systems in this series of SMAs, a Ni-rich specimen Ti_49_Ni_45_Fe_6_ was fabricated. Remarkably, a slight change in Ti/Ni ratio converts Ti_49_Ni_45_Fe_6_ SMA into a strain glass system. Overall, the evolution of phase transformation in the Fe-substituted TiNi SMAs is presumably caused by the changes in local lattice structure via the induced local strain fields by Fe point defects.

## Introduction

The TiNi-based shape memory alloys (SMAs) are one of the most studied functional materials because they have superior properties such as thermal and mechanical memory^1–4^. For instance, an equiatomic TiNi (Ti_50_Ni_50_) alloy undergoes a first-order phase transition from the cubic austenite phase (B2) to a monoclinic martensite phase (B19′) during the cooling process. In general, the martensite state is defined as the long-range strain-ordered ferroelastic state below martensitic transition^5^. That is, the martensite state is analogous to the long-range ordering of electric dipoles and magnetic spins in ferroelectrics and ferromagnets, respectively^6,7^. It is well-established that the introduction of point defects plays an important role in modifying and controlling the properties of ferroelastics^4,8^. In addition to the two normal states of austenite (paraelastic) and martensite (ferroelastic), point defects in the SMAs can create two abnormal states such as precursor state and strain-glass state in the ferroelastic system^9–14^. These abnormal states are referred as the non-ideal strain states of ferroelastics^13^. Notably, the precursor state has been studied extensively in recent years^10–18^. This is because the precursor state is neither a fully disordered strain state like austenite nor a fully ordered strain state like martensite^13,15,16^. However, the precursor state is ergodic, although it is not a frozen glass state^18^. On the other hand, strain glass is known as a frozen-disordered strain state caused by fluctuations of randomly distributed point defects.

Typically, point defects are induced via alloying/substituting of equiatomic TiNi SMA with various elements including Fe, Co, Cr, Mn, Ni, and Cu^4,11–13,19–26^. All these substituents (except Cu) induce the R-martensite ordering into the TiNi SMA together with a complete suppression of martensite B19′ phase. Most importantly, the strain glass features emerge in these substituted SMAs when the substitution exceeds a critical concentration, *x*_*c*_. Particularly, the Co-substituted TiNi SMAs has a higher *x*_*c*_ value (~9 at.%) than that of other substituted SMAs (≤6 at.%). The *x*_*c*_ values are about 6.0, 4.5, and 5.5 at.% for the Fe-, Cr-, and Mn-substituted TiNi SMAs, respectively^19,20,22–24^. However, excess Ni content has a strong influence on the martensitic transformation features of Ti_50-*x*_Ni_50+*x*_ SMA, as the martensitic transition remains intact for *x* < 1.3 and the martensitic transition is suppressed for *x* ≥1.3^23,26^. In our earlier works^27,28^, we showed that low-*T* aging/annealing also results in a significant change in the martensitic transformation features of Ti_48.7_Ni_51.3_ SMA. That is, the strain glass order in as-quenched Ti_48.7_Ni_51.3_ is transformed to the R-martensite order by the aging process^27^. This is mainly due to the local lattice deformations which induced by the aging created Ti_3_Ni_4_ precipitates. Interestingly, the element Cu has a unique nature that it can be substituted for Ni up to 30 at.% and still exhibits shape memory effects^24,25^. Recently, Frenzel *et al*.^24^ observed a linear variation of martensitic transition temperature (*M*_*s*_) in the Ti_50_Ni_45_Cu_5_ SMA with a small variation in Ni content. This work confirmed that the stoichiometry of Ni atoms also plays a key role in the martensitic transformation of the substituted TiNi SMAs^4,23,24,26^.

Among the third element substituted TiNi systems, the Fe-substituted TiNi (Ti_50_Ni_50-*x*_Fe_*x*_) SMAs are extremely interesting materials for fundamental and applied research^4,11–15,19,20,23,25^. Remarkably, a generic phase diagram using the data of the Ti_50_Ni_50-*x*_Fe_*x*_ SMAs has been proposed to describe the relationships among all strain states in ferroelastics^13,20^. That is, the Ti_50_Ni_50-*x*_Fe_*x*_ SMAs have a two-step martensitic transformation (B2 → R and R → B19′) below *x* = 3.0, while a one-step R-phase transformation (B2 → R) is observed for 3.0 ≤ *x* ≤ 5.0. For *x* ≥ 6.0, strain glass features emerge^13,20^. A recent calculation by Niu and Geng using the density functional theory showed that the substitution of Fe into the TiNi lattice induces anti-precursor effects^29^. This is because the Fe atom triggers a drastic atomic-scale local lattice distortion, which leads to the intermediate structure between the B2 and R phases. As a result, the formation of precipitates is unlikely in the Ti_50_Ni_1-*x*_Fe_*x*_ SMAs^29^.

Moreover, a study by Wang *et al*.^30^ revealed that the non-martensitic Ti_48.5_Ni_51.5_ alloy (which goes through the strain glass transition) has both shape memory effect and superelasticity. These effects have developed primarily due to the stress-induced transformation from a short-range strain-state to a long-range strain-ordered martensite and vice versa. Therefore, it will be very informative to carry out a thorough study on the transport and thermal properties of the Fe-substituted TiNi SMAs, Ti_50_Ni_50-*x*_Fe_*x*_. In particular, the highly sensitive Seebeck coefficient and thermal conductivity measurements on the Ti_50_Ni_50-*x*_Fe_*x*_ SMAs will be crucial to explore their thermal transport properties, which have not yet been fully explored. In this respect, we recently reported the electrical and thermal transport properties of the Ti_50_Ni_48.5_Fe_1.5_ and Ti_50_Ni_46_Fe_4_ SMAs^27,31^. From these studies, it was shown that both the Fermi energy (*E*_*F*_) and the density of states (DOS) of TiNi SMAs vary considerably with the Fe concentration (*x*). In addition, the phonon-electron coupling is noticeably weakened by Fe substitution, which leads to the change in the transformation features of these SMAs^27,31^.

With the background of these earlier works, we performed a comprehensive study on the transport and thermal properties of the Ti_50_Ni_50-*x*_Fe_*x*_ (*x* = 2.0–10.0) SMAs to investigate the impact of Fe substitution on the characteristics of martensitic transformation in TiNi SMA. In the study, we observed that the Ti_50_Ni_48_Fe_2_ and Ti_50_Ni_47_Fe_3_ SMAs display a two-step martensitic transformation (B2 → R and R → B19′), while the Ti_50_Ni_50-*x*_Fe_*x*_ SMAs with *x* = 4.0–6.0 have a one-step transition (B2 → R). However, the characteristics of strain glass transition are seen for the Ti_50_Ni_42_Fe_8_ and Ti_50_Ni_40_Fe_10_ SMAs. Most importantly, we decisively confirm that Ti_50_Ni_44_Fe_6_ is the boundary composition for the studied Ti_50_Ni_50-*x*_Fe_*x*_ SMAs, which dividing the martensite (*x* ≤ 6.0) and strain glass (*x* > 6.0) state SMAs. Here, a careful comparison of the crossover composition SMA Ti_50_Ni_44_Fe_6_ with a slightly Ni-rich alloy Ti_49_Ni_45_Fe_6_ was made. Markedly, a small amount of excess Ni transforms the martensitic R-phase transition in Ti_50_Ni_44_Fe_6_ to a strain glass transition in Ti_49_Ni_45_Fe_6_. Such an observation suggests that the excess Ni atoms are likely occupying (as antisite defects) the vacancy sites of Ti atoms, as in the case of Ni-rich Ti_50-*x*_Ni_50+*x*_ SMAs.

## Results

### Electrical resistivity

**Figure 1:**
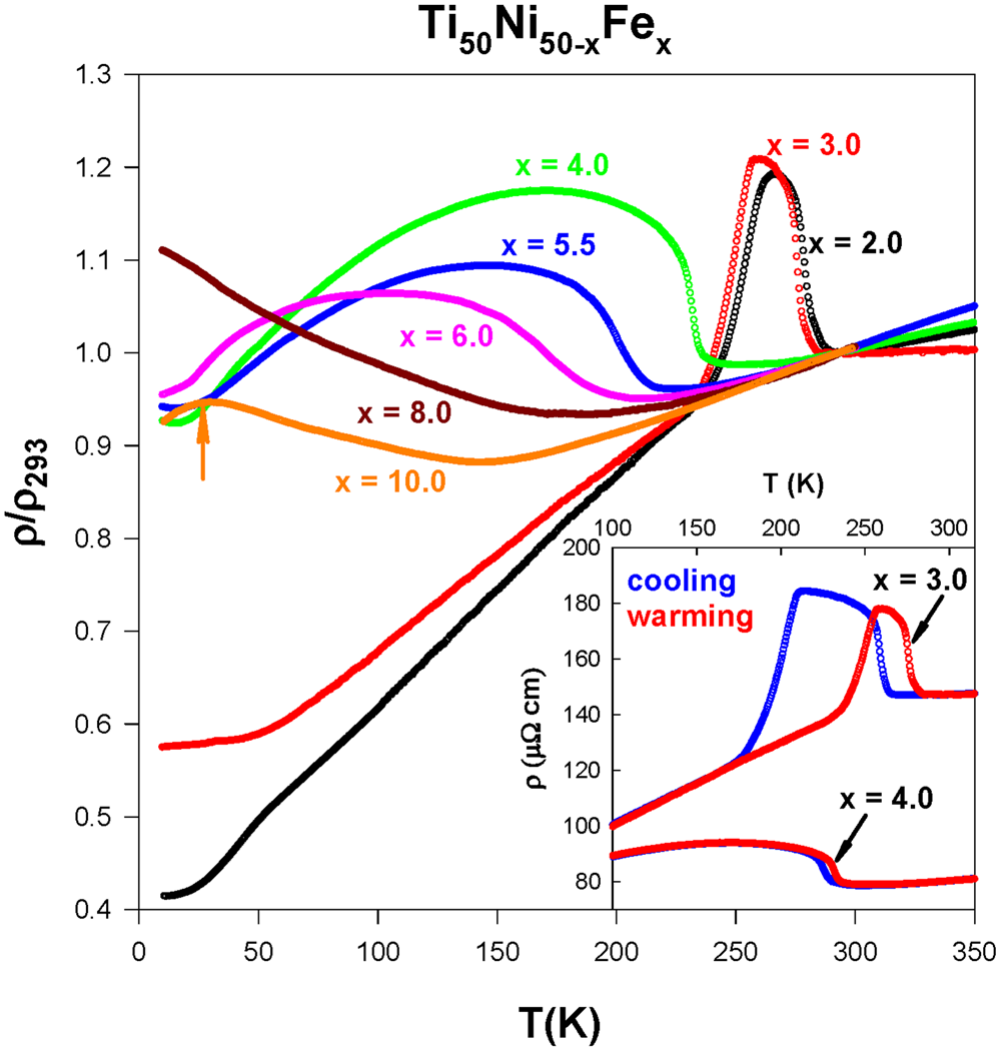
The normalized electrical resistivity, *ρ*(*T*)/*ρ*_293K_ of the Ti_50_Ni_50-*x*_Fe_*x*_ (*x* = 2.0–10.0) SMAs measured during the warming cycle and the normalization is done with the division of the measured *ρ*(*T*) by the resistivity value at 293 K, *ρ*_293K_. The inset shows the *ρ*(*T*) data of the Ti_50_Ni_47_Fe_3_ and Ti_50_Ni_46_Fe_4_ SMAs in the cooling and warming cycles.

Figure 1 displays the normalized electrical resistivity, *ρ*(*T*)/*ρ*_293K_ versus temperature for the Ti_50_Ni_50-*x*_Fe_*x*_ (*x* = 2.0–10.0) SMAs, which measured during the warming cycle. It is noted that the Ti_50_Ni_48_Fe_2_ and Ti_50_Ni_47_Fe_3_ SMAs showed a two-step martensitic transformation^19,32^. Especially, the Ti_50_Ni_47_Fe_3_ SMA has a noticeable thermal hysteresis temperature (Δ*T*_*H*_) of about 15 K and 45 K for the transitions B2 → R and R → B19′, respectively (see inset of Fig. 1). In contrast, the Ti_50_Ni_46_Fe_4_ and Ti_50_Ni_44.5_Fe_5.5_ SMAs displayed a one-step R-phase martensitic transition (B2 → R) with a much smaller Δ*T*_*H*_ value of less than 6 K, while Δ*T*_*H*_ is negligible (less than 1 K) for Ti_50_Ni_44_Fe_6_. This observation is in agreement with the literature that the one-step R-phase transition in the TiNi-based SMAs has a less pronounced thermal hysteretic behavior than that of the two-step one^27^. However, the Ti_50_Ni_50-*x*_Fe_*x*_ SMAs with *x* > 6 (Ti_50_Ni_42_Fe_8_ and Ti_50_Ni_40_Fe_10_) show the features of strain glass (short-range strain order)^19,20^.

Using the resistivity data, the characteristic temperatures such as the martensitic start temperature *M*_*s*_ and the *T*_*min*_ (the temperature at which the resistivity minimum occurs) of the strain glass compounds are obtained and listed in Table 1. These data showed that the characteristic temperature decreases with an increasing Fe content (*x*), which is consistent with the literature^19,20^. In particular, the *M*_*s*_ value decreases considerably with *x* > 3.0. In addition, the transformation width (Δ*T*) of the B2 → R transition is estimated to be above 70 K for *x* > 3.0 (Table 1), much larger than the Ti_50_Ni_48_Fe_2_ and Ti_50_Ni_47_Fe_3_ SMAs (<30 K). The pronounced change in characteristic temperature and transformation width with Fe substitution (*x* > 3.0) can be clearly seen in the inset of Fig. 1. Such a finding demonstrates that the Fe substitution into the Ni sites of TiNi SMA has a strong influence on the characteristics of martensitic transformation when the substitution level is beyond *x* = 3.0^24^.

**Table 1:** The deduced values of the characteristic temperatures (*M*_*s*_ and *T*_*min*_), transformation width (Δ*T*) of the B2 → R transition, the Fermi energy (*E*_*F*_), and the enthalpy change (Δ*H*) during the martensitic transformation for the Ti_50_Ni_50-*x*_Fe_*x*_ (*x* = 2.0–10.0) and Ti_49_Ni_45_Fe_6_ samples.

Sample	*M*_*s*_ and *T*_*min*_ (K) from the *ρ*(*T*)	Δ*T* (K) of the B2 → R transition from the *ρ*(*T*)	*M*_*s*_ (K) from the *S*(*T*)	*E*_*F*_ (eV)	Δ*S* (J/mole K)	Δ*H* (J/g)
Ti_50_Ni_48_Fe_2_	293.1	25.1	288.2	2.4	1.31	18.1
Ti_50_Ni_47_Fe_3_	285.5	27.2	280.4	3.9	1.37	17.3
Ti_50_Ni_46_Fe_4_	250.9	76.3	236.8	1.9	1.35	6.1
Ti_50_Ni_44.5_Fe_5.5_	225.6	77.9	205.0	2.2	1.28	3.6
Ti_50_Ni_44_Fe_6_	209.1	108.4	176.3	2.3	0.32	1.6
Ti_49_Ni_45_Fe_6_	123.5	—	—	2.4	—	—
Ti_50_Ni_42_Fe_8_	185.7	—	—	2.7	—	—
Ti_50_Ni_40_Fe_10_	142.0	—	—	3.2	—	—

Upon Fe substitution, the value of *ρ*_293K_ decreases substantially above *x* = 3.0. For example, the *ρ*_293K_ value decreases to <90 μΩ cm (for *x* ≥ 4.0) from about 147 μΩ cm of Ti_50_Ni_47_Fe_3_. However, it becomes nearly constant above *x* = 6.0, i.e. their *ρ*_293K_ values lie in the range of 67.0–70.0 μΩ cm. However, the low-*T* resistivity (*ρ*_10K_) increases with Fe content up to *x* = 8.0 and then it decreases for *x* = 10.0. Surprisingly, the Ti_50_Ni_40_Fe_10_ SMA displays a positive temperature coefficient of resistivity below 30 K, which shown by an arrow in Fig. 1. This feature of Ti_50_Ni_40_Fe_10_ may be attributed to the induced metallic character by heavy Fe substitution. This will be further examined using the highly sensitive Seebeck coefficient, which presented in the Seebeck coefficient section.

**Figure 2:**
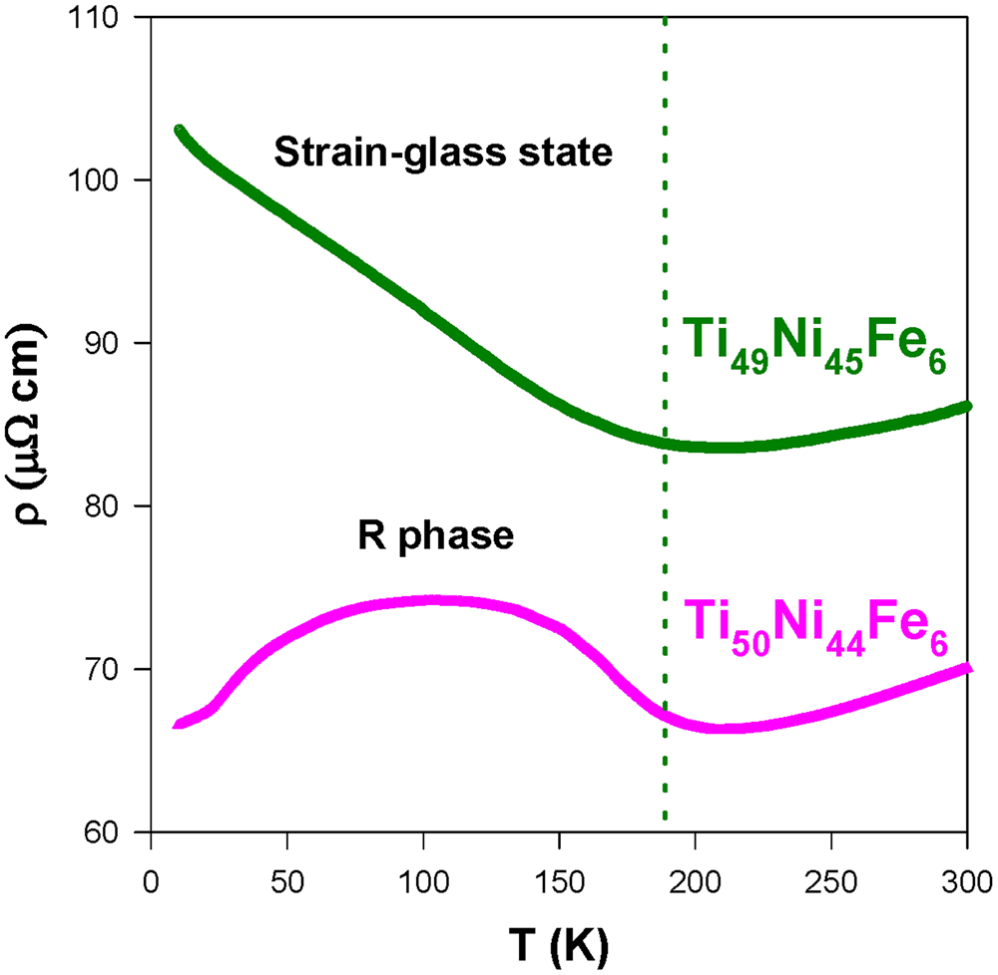
The measured *ρ*(*T*) data of the Ti_50_Ni_44_Fe_6_ and Ni-rich Ti_49_Ni_45_Fe_6_ compounds during the warming cycle. The dotted line is drawn roughly to show the different low-*T* states for these samples.

**Figure 3:**
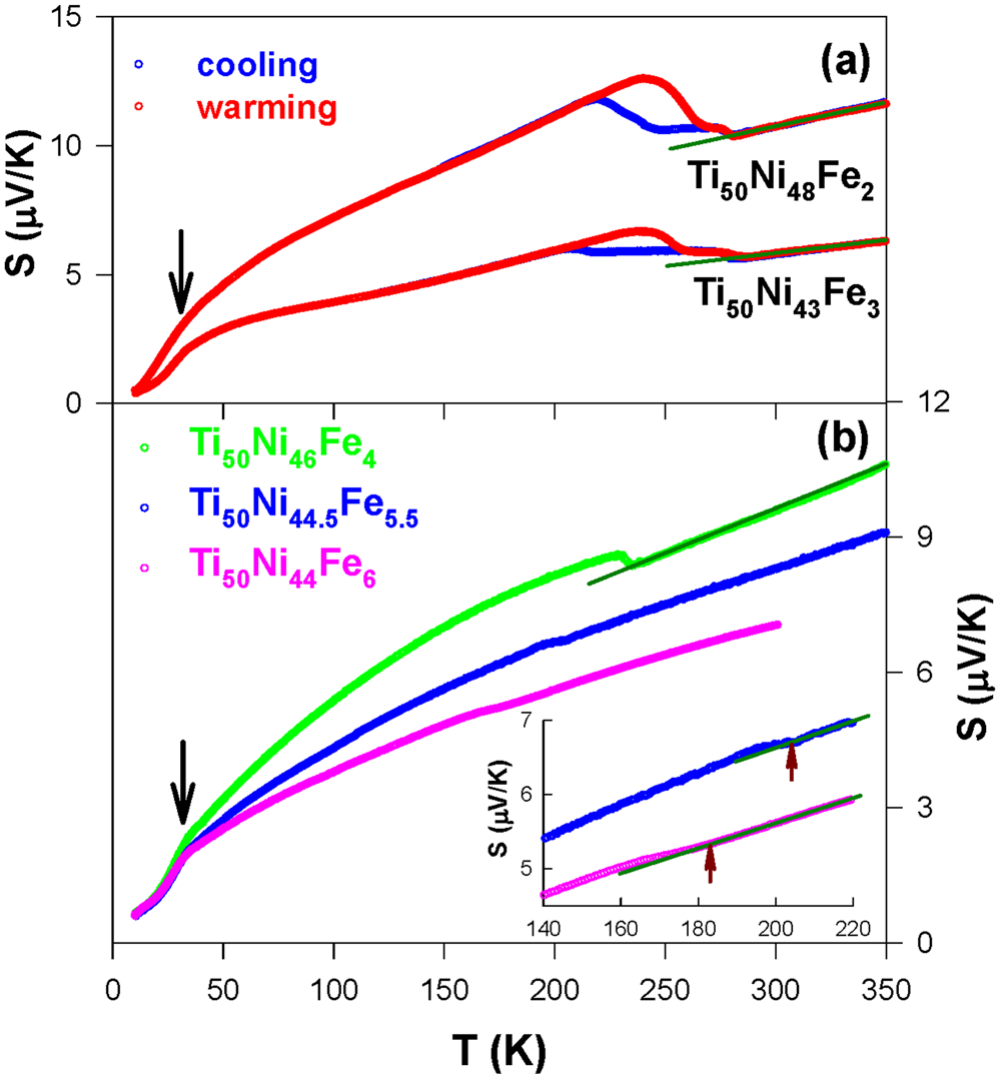
(**a**) The Seebeck coefficient, *S*(*T*) data of the Ti_50_Ni_48_Fe_2_ and Ti_50_Ni_47_Fe_3_ SMAs in the cooling and warming cycles, and (**b**) the *S*(*T*) data of Ti_50_Ni_46_Fe_4_, Ti_50_Ni_44.5_Fe_5.5_, and Ti_50_Ni_44_Fe_6_ SMAs in the warming cycle. Inset of Fig. 3b displays the *S*(*T*) data of the Ti_50_Ni_44.5_Fe_5.5_ and Ti_50_Ni_44_Fe_6_ SMAs near the R-phase transformation. The solid lines represent the linear fits to the high-*T S*(*T*) data of the samples using the Mott’s equation.

**Figure 4:**
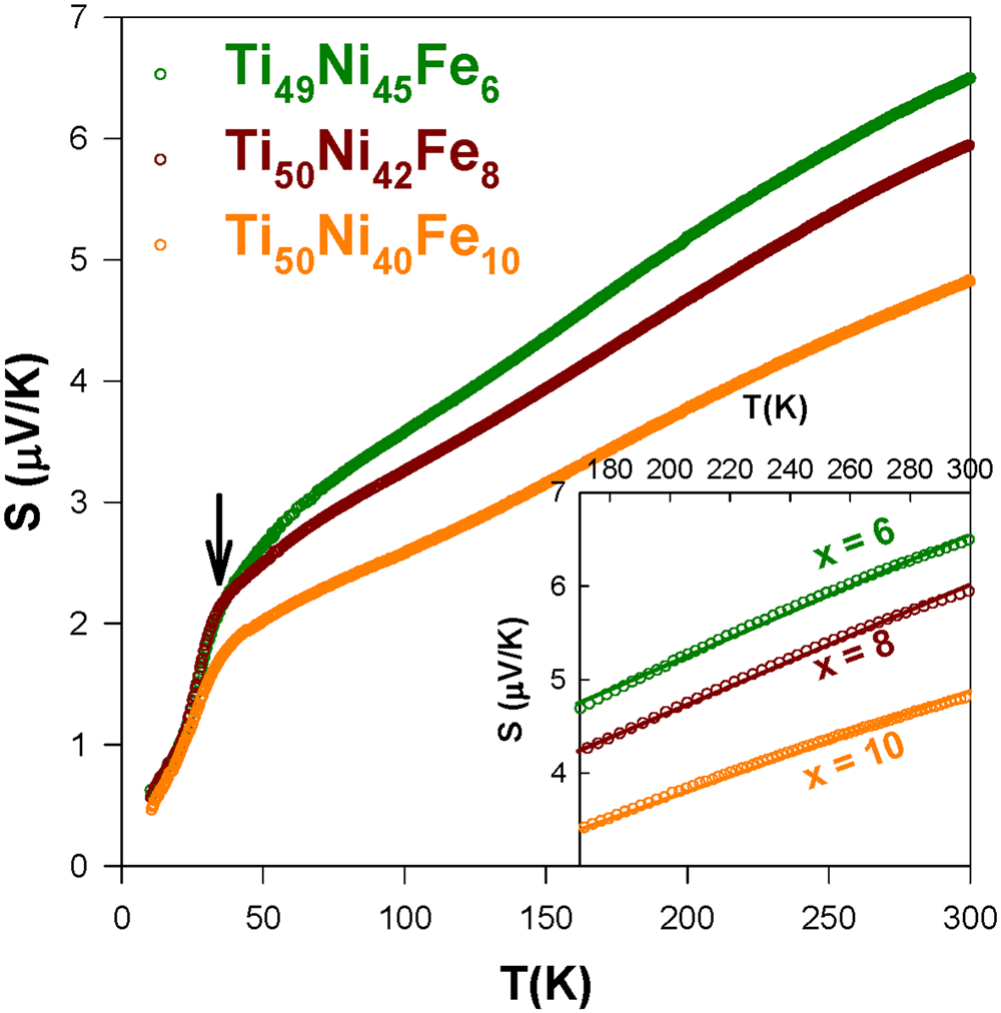
The measured *S*(*T*) data of the Ti_49_Ni_45_Fe_6_, Ti_50_Ni_42_Fe_8_, and Ti_50_Ni_40_Fe_10_ alloys in the warming cycle. The inset shows the high-*T S*(*T*) data of these alloys and the solid lines denote the corresponding linear fits.

We also investigated the Ni-rich Fe-substituted SMA Ti_49_Ni_45_Fe_6_ to explore the influence of excess Ni on the boundary composition (*x* = 6) of the Ti_50_Ni_50-*x*_Fe_*x*_ system^19,20^. In fact, Wang *et al*. showed the existence of strain glass beyond a critical content of 5.0 < *x*_*c*_ < 6.0^20^. Here, we plotted the resistivity data of Ti_49_Ni_45_Fe_6_ SMA together with the Ti_50_Ni_44_Fe_6_ SMAs in Fig. 2 to a compare the nature of their strain state ordering. Evidently, a slight excess Ni (about 1 at.%) in Ti_49_Ni_45_Fe_6_ SMA has transformed the system to a short-range strain ordering from the long-range R-martensite ordering of the Ti_50_Ni_44_Fe_6_ SMA. This observation reveals that the Ni/Ti ratio also plays a key role in determining the critical content, *x*_*c*_. Interestingly, the studied strain glass alloys (Ti_49_Ni_45_Fe_6_, Ti_50_Ni_42_Fe_8_, and Ti_50_Ni_40_Fe_10_) in the present work have a positive and negative temperature coefficient of resistivity at high and low temperatures (see Figs 1 and 2), respectively. However, this observation is distinct from the positive temperature coefficient behavior over the entire temperature range of 10–300 K for the Ni-rich Ti_50-*x*_Ni_50+*x*_ (*x* ≥ 1.3) SMAs^26^. Besides, we estimated the inflection point (*T*_0_) using the *ρ*(*T*) data for two strain glass samples Ti_50_Ni_40_Fe_10_ and Ti_49_Ni_45_Fe_6_ of the present work and their *T*_0_ values are about 92 and 124 K, respectively. This finding indicates that the strain-glass transition temperature of these Fe-substituted TiNi SMAs is much lower than the Ni-rich SMAs Ti_48.7_Ni_51.3_ and Ti_48.4_Ni_51.6_ (>175 K)^26^. In order to further explore the martensitic transformation features of these Fe-substituted TiNi SMAs, we carried out the Seebeck coefficient measurement (see Figs 3 and 4). The Seebeck coefficient is a highly sensitive probe for the phenomenon that involves the changes in Fermi level DOS such as the phase transition. Recently, Kustov *et al*.^33^ revealed that the estimation of the exact value of martensitic temperatures (*M*_*S*_) using the resistivity data is rather difficult, due to the anelastic effects during the heating and cooling cycles. Hence, the Seebeck coefficient measurement may provide an alternative way to determine *M*_*S*_ with better accuracy.

### Seebeck coefficient

The temperature-dependent Seebeck coefficient *S*(*T*) of Ti_50_Ni_50-*x*_Fe_*x*_ (*x* = 2.0–6.0) SMAs is presented in Fig. 3. It is observed that Ti_50_Ni_48_Fe_2_ and Ti_50_Ni_47_Fe_3_ SMAs display a pronounced two-step martensitic transformation above 250 K with a noticeable thermal hysteresis (Fig. 3a). Whereas, a step-like feature or a slope change was observed below 250 K for the Ti_50_Ni_50-*x*_Fe_*x*_ SMAs with *x* = 4.0–6.0 (Fig. 3b), which is associated to the R → B2 transition. Notably, the transition temperature *M*_*s*_ decreases noticeably with Fe content above *x* = 3.0 (Table 1). These observations are consistent with the resistivity data. However, we noticed that the estimated *M*_*s*_ values for these SMAs from the *S*(*T*) data are lower than the values that deduced from the resistivity data. This finding suggests that the transition temperature (*M*_*s*_) of these SMAs in the resistivity data is slightly shifted to a higher temperature than its actual value. This is presumably due to the structural anelasticity of the TiNi-based SMAs during the thermal cycles^33^. In addition, the Seebeck coefficient measurement is not as sensitive to the grain boundaries and defects as the resistivity measurement^26^. Thus, we argue that the Seebeck coefficient measurement is a more reliable probe for the evaluation of the martensitic temperatures of the TiNi-based SMAs.

Conversely, no noticeable anomalous features can be detected in the *S*(*T*) data for the samples Ti_49_Ni_45_Fe_6_, Ti_50_Ni_42_Fe_8_, and Ti_50_Ni_40_Fe_10_ (Fig. 4), similar to the behavior of the Ni-rich Ti_48.7_Ni_51.3_ and Ti_48.4_Ni_51.6_ SMAs^26^. In addition, all the samples show positive *S* values over the entire temperature range, indicating that the dominant charge carriers are holes (*p*-type carriers) in the Ti_50_Ni_50-*x*_Fe_*x*_ systems. It is worth mentioning here that the excess Fe point defects alter the strain state of Ti_50_Ni_42_Fe_8_ and Ti_50_Ni_40_Fe_10_ alloys to short-range strain (strain-glass) state from the R-martensite state of Ti_50_Ni_44_Fe_6_ SMA, while the excess Ni atoms at Ti-sites induce the strain glass ordering in the Ni-rich alloy Ti_49_Ni_45_Fe_6_ from that of same SMA Ti_50_Ni_44_Fe_6_.

Below the transition temperature, the *S*(*T*) data of the Ti_50_Ni_50-*x*_Fe_*x*_ SMAs show a typical metallic diffusive behavior. Particularly, a hump-like feature below 40 K was observed as a result of the phonon-drag effect (indicated by the arrows in Figs 3 and 4)^25–27^. At high temperatures above the transition, the measured *S*(*T*) varies rather linearly with temperature. This is generally observed for the TiNi-based SMAs with the diffusive thermoelectric transport^25–27,31^. That is, the linear variation of *S* with temperature is expected for metals according to Mott’s formula, $S=\frac{{\pi }^{2}{k}_{B}^{2}}{2e{E}_{F}}T=bT$, where *k*_*B*_ is the Boltzmann constant and *E*_*F*_ is the Fermi energy. The linear fits to the high-*T S*(*T*) data of the Ti_50_Ni_50-*x*_Fe_*x*_ SMAs using Mott’s equation are illustrated as solid lines in Figs 3 and 4, and the deduced *E*_*F*_ values are given in Table 1. We found a significant decrease in the *E*_*F*_ value from Ti_50_Ni_47_Fe_3_ to Ti_50_Ni_46_Fe_4_, possibly due to the different type of transition which may alter the DOS near Fermi level. In addition, the *E*_*F*_ value increases substantially with *x* > 6.0 as the samples enter the strain glass state. This may also relate to the change of the transition nature together with a higher content Fe substitution. In general, the point defect (Fe) induces a noticeable change in the *E*_*F*_ and the Fermi level DOS of TiNi SMA^25–27^. Hence, the characteristics of martensitic transformation in TiNi SMA are altered considerably with Fe substitution (see Table 1). Besides, the Ni-rich Ti_49_Ni_45_Fe_6_ has a comparable *E*_*F*_ value (∼2.4 eV) to that of Ti_50_Ni_44_Fe_6_ (*E*_*F*_ ∼ 2.3 eV), although they show different types of transition at low temperatures. Such a behavior indicates that there could be other factors such as Ni antisite defects also affecting the DOS near Fermi level in the Fe-substituted TiNi SMAs, which warrants further investigation.

### Thermal conductivity

**Figure 5:**
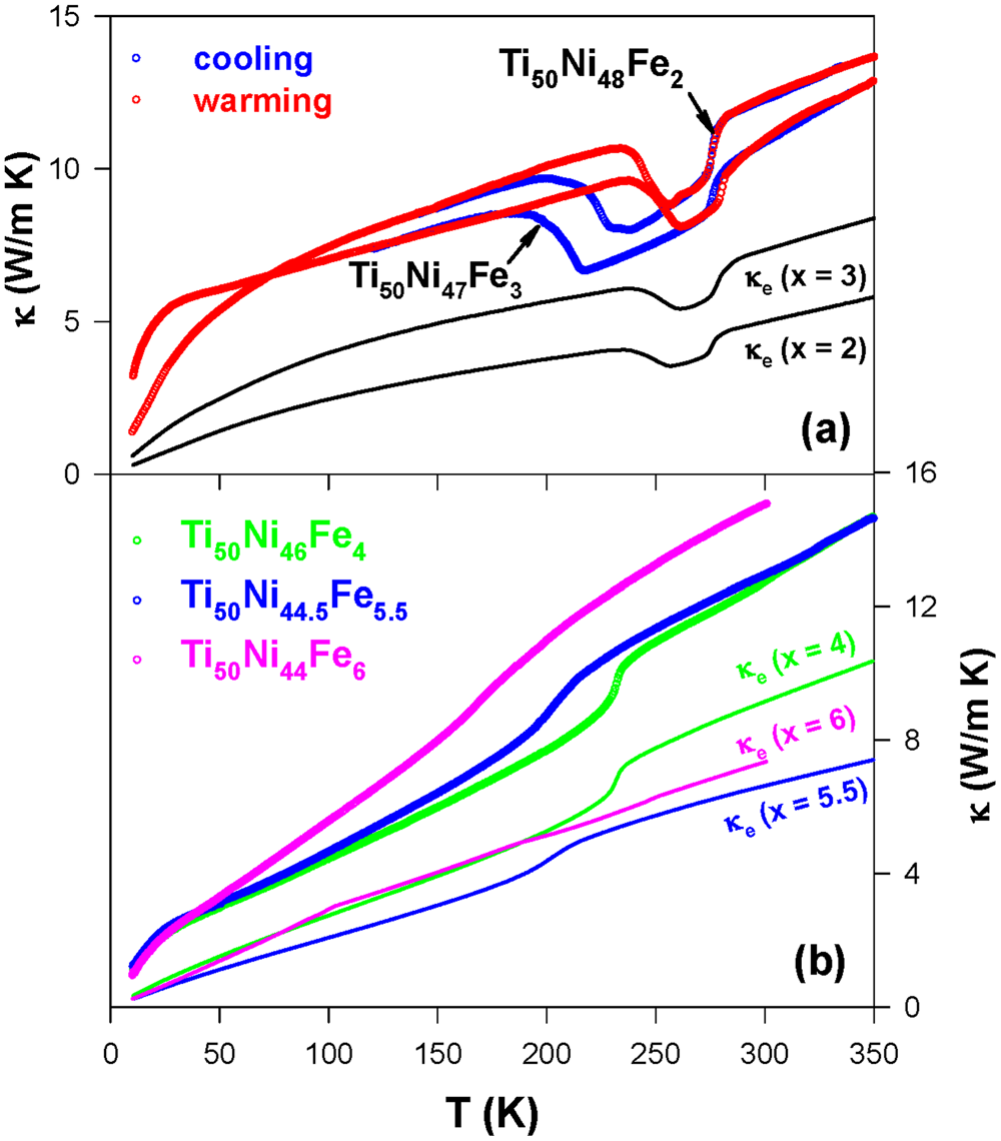
The measured thermal conductivity *κ*(*T*) of (**a**) Ti_50_Ni_48_Fe_2_ and Ti_50_Ni_47_Fe_3_ SMAs, and (**b**) Ti_50_Ni_46_Fe_4_, Ti_50_Ni_44.5_Fe_5.5_, and Ti_50_Ni_44_Fe_6_ alloys in the warming process. The solid lines illustrate the electronic thermal conductivity *κ*_*e*_(*T*) of these alloys.

**Figure 6:**
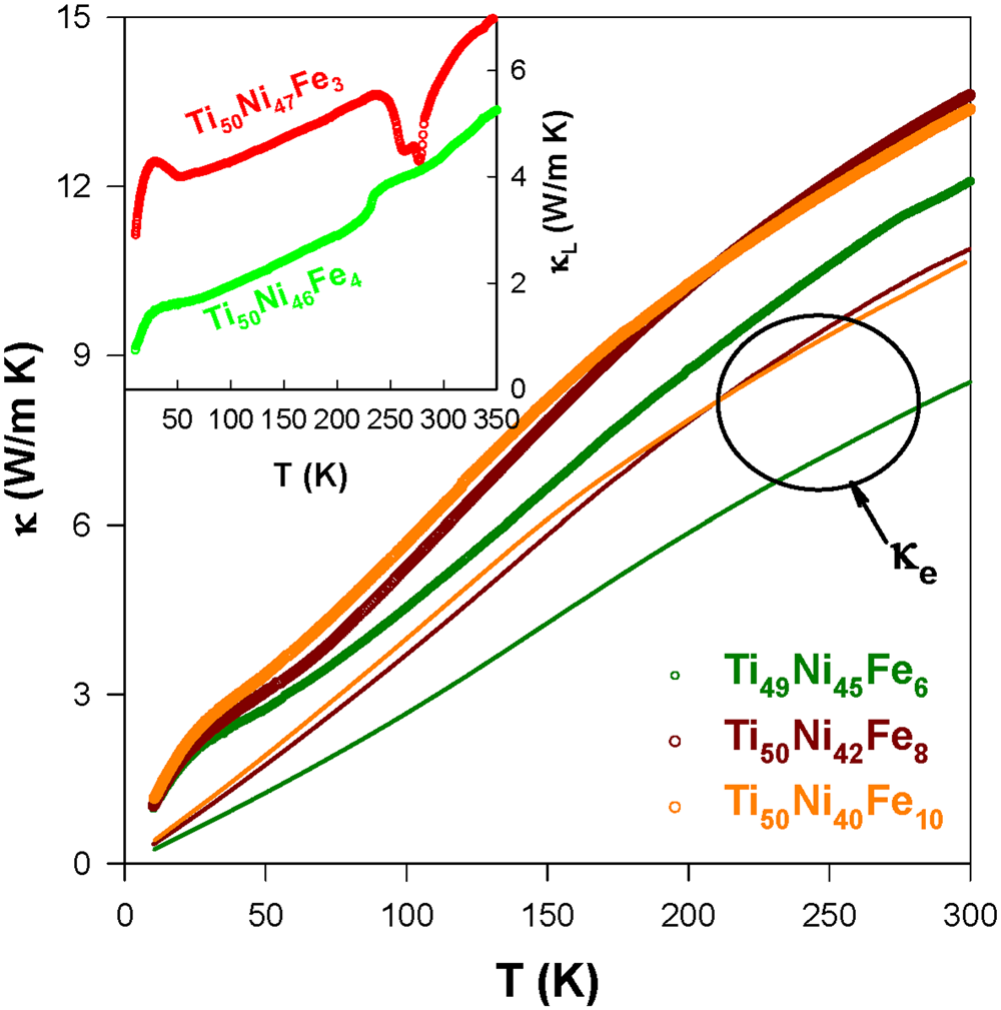
The measured *κ*(*T*) data of Ti_49_Ni_45_Fe_6_, Ti_50_Ni_42_Fe_8_, and Ti_50_Ni_40_Fe_10_ alloys during the warming cycle and the solid lines correspond to their *κ*_*e*_(*T*). The inset displays the lattice thermal conductivity *κ*_*L*_(*T*) data of the Ti_50_Ni_47_Fe_3_ and Ti_50_Ni_46_Fe_4_SMAs, estimated using the Wiedemann-Franz law.

The thermal conductivity *κ*(*T*) of the Ti_50_Ni_50-*x*_Fe_*x*_ (*x* = 2.0–6.0) SMAs during the warming cycle is illustrated in Fig. 5. It is noted that Fe substitution induces a reduction in the RT thermal conductivity (*κ*_RT_ ∼ 11.0–15.0 W/m K) as compared to parent Ti_50_Ni_50_ (*κ*_RT_ ∼ 17.0 W/m K)^26^. The Ti_50_Ni_50-*x*_Fe_*x*_ SMAs with *x* = 2.0–6.0 display a step-like feature near the martensitic transition^27,32^. However, the strength of the step-like feature diminishes progressively with increasing *x*. However, this feature is completely different from a spike-shaped anomaly in the Ti_50_Ni_50_ and Ti_50_Ni_48.5_Fe_1.5_ SMAs^26,31^. This finding validates that the electron-phonon coupling near the martensitic transition is weakened by Fe substitution in the TiNi SMAs with *x* ≥ 2.0 that we examined here. This is due to the induced local lattice distortions by Fe point defects in the TiNi lattice^27^. Whereas the Ti_50_Ni_42_Fe_8_, Ti_50_Ni_40_Fe_10_, and Ti_49_Ni_45_Fe_6_ alloys do not show any anomalous features in the measured *κ*(*T*) data (Fig. 6), similar to the behavior of the strain glass Ni-rich TiNi SMAs^26^.

It is well-known that thermal conductivity measurements can give valuable information about various scattering processes of thermal carriers that are involved in solids. Hence, it is important to probe the role of charge and phonon carriers on the heat conduction of the Ti_50_Ni_50-*x*_Fe_*x*_ SMAs. For the metallic compounds, the total thermal conductivity can be divided into the electronic thermal conductivity (*κ*_*e*_) and the lattice thermal conductivity (*κ*_*L*_). The electronic thermal conductivity *κ*_*e*_(*T*) of the studied SMAs is estimated by using the Wiedemann-Franz law: *κ*_*e*_ *ρ*/*T* = *L*_0_, where *L*_0_ (=2.45 ×10^−8^ WΩK^−2^) is the Lorenz number, and the results are shown as solid lines in Fig. 5. The lattice thermal conductivity *κ*_*L*_(*T*) is then obtained by subtracting the *κ*_*e*_(*T*) from the measured *κ*(*T*) (see the inset of Fig. 6 for representative samples). It is clear from this estimation that *κ*_*e*_ contributes more than half of the total *κ* for all studied samples at high temperatures (especially in the B2 phase), similar to the behavior of Cu- and Ni-substituted TiNi SMAs^25–27^. For instance, the contribution of *κ*_*e*_ to total *κ* at 293 K increases considerably from less than 50% of the Ti_50_Ni_47_Fe_3_ SMA to larger than 60% for *x* ≥ 4.0 (Figs 5 and 6). Particularly, the *κ*_*e*_ donates about 80.0% to total *κ* of the samples Ti_50_Ni_42_Fe_8_ and Ti_50_Ni_40_Fe_10_ at 293 K. This finding validates the complete weakening of the phonon-electron coupling in the TiNi-based SMA when Fe substituted beyond *x* = 6.0. Besides, two transitions (B2 → R and R → B19′) are clearly seen in the *κ*_*L*_(*T*) data of the Ti_50_Ni_48_Fe_2_ and Ti_50_Ni_47_Fe_3_ SMAs (see inset of Fig. 6). Remarkably, it is clearly seen in the inset of Fig. 6 that a slight increase of Fe content from *x* = 3.0 to *x* = 4.0 leads to a significant change in the martensitic transformation from a two-step transformation to a one-step transition (B2 → R).

It is obvious that the observed R-phase transition in the Ti_50_Ni_44_Fe_6_ SMA is mainly due to electronic contribution, as the slope change near the transition is much pronounced in the *κ*_*e*_(*T*) data than its *κ*_*L*_(*T*). However, the sample Ti_49_Ni_45_Fe_6_ does not show the noticeable anomalous feature in *κ*, presumably due to the excess Ni atoms that occupy the vacancy Ti sites as antisite defects^26^. Overall, a gradual weakening of electron-phonon coupling of the TiNi SMA occurs upon Fe substitution until *x* = 6.0, and finally, the coupling disappears completely beyond *x* = 6.0. Hence, no anomalous features are seen for the Ti_50_Ni_50-*x*_Fe_*x*_ SMAs with *x* > 6.0. In addition, the introduction of a slight excess Ni into the Ti sites in Ti_50_Ni_44_Fe_6_ also weakens the electron-phonon coupling drastically in the compound Ti_49_Ni_45_Fe_6_, as the R-phase transition in Ti_50_Ni_44_Fe_6_ is completely suppressed.

### Specific heat

**Figure 7:**
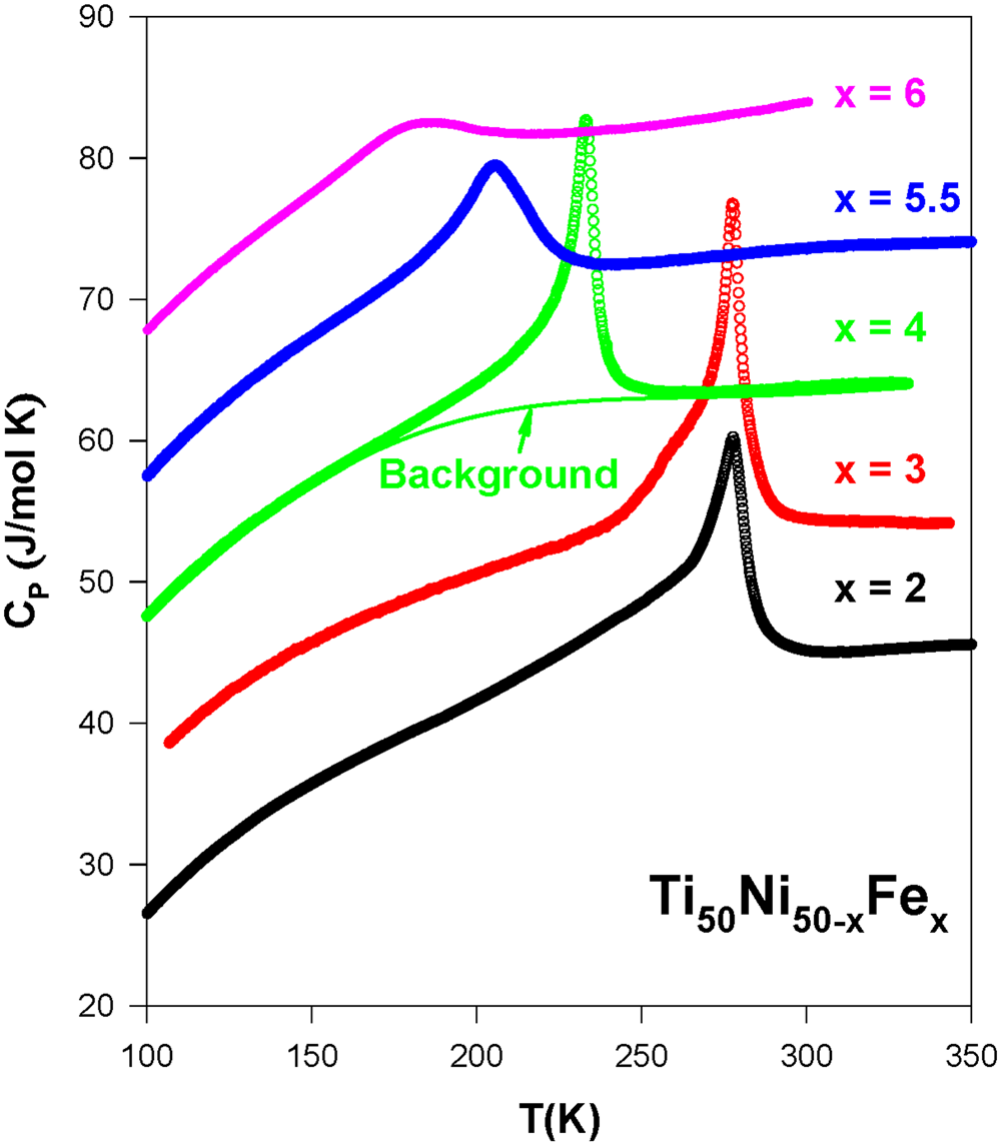
The specific heat versus temperature, *C*_*P*_(*T*) for the Ti_50_Ni_50-*x*_Fe_*x*_ (*x* = 2.0–6.0) SMAs and the solid line illustrates a smooth lattice background to the *C*_*P*_(*T*) data of the Ti_50_Ni_46_Fe_4_. The curves have been offset vertically for clarity.

**Figure 8:**
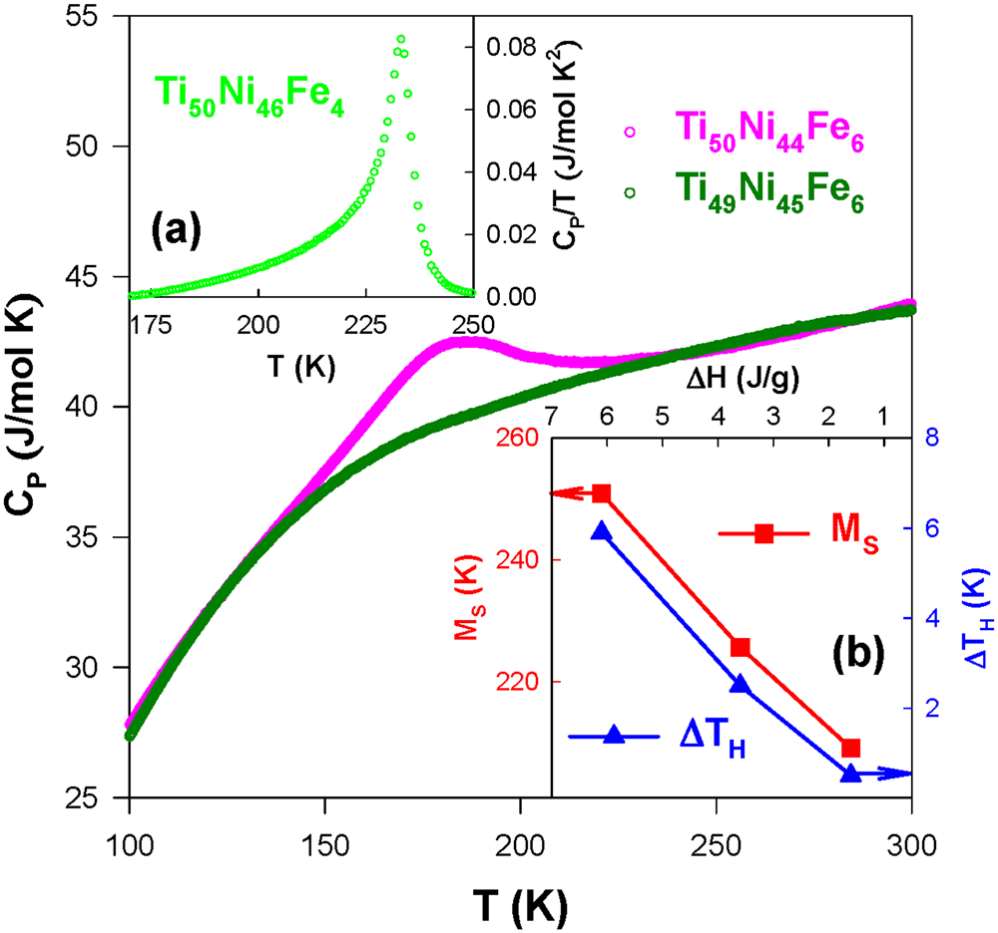
The comparison of *C*_*P*_(*T*) data of Ti_50_Ni_44_Fe_6_ with a Ni-rich alloy Ti_49_Ni_45_Fe_6_. The inset (**a**) displays the *C*_*P*_/*T* versus *T* for the Ti_50_Ni_46_Fe_4_ SMA, and the inset (**b**) shows the plots of the transition temperature (*M*_*s*_) and width of thermal hysteresis (Δ*T*_*H*_) versus the enthalpy change (Δ*H*) across the R-phase transition for the Ti_50_Ni_50-*x*_Fe_*x*_ (*x* = 4.0–6.0) SMAs.

Figure 7 displays the temperature-dependent specific heat *C*_*P*_(*T*) of the Ti_50_Ni_50-*x*_Fe_*x*_ (*x* = 2.0–6.0) SMAs during the warming cycle. The Ti_50_Ni_48_Fe_2_, Ti_50_Ni_47_Fe_3_, and Ti_50_Ni_46_Fe_4_ SMA samples have sharp transition features near the martensitic transition^27,31^. However, it is noted that the magnitude of the peak decreases gradually with *x* > 3.0^19^. The two-step transitions (B2 → R and R → B19′) are clearly visible for the Ti_50_Ni_47_Fe_3_ SMA, as seen in its *κ*_*L*_(*T*) data (see inset of Fig. 6). In contrast, the slightly Ni-rich sample Ti_49_Ni_45_Fe_6_ does not show any detectable anomalous features associated with the strain glass transition in the *C*_*P*_(*T*) when compared to the Ti_50_Ni_44_Fe_6_ SMA (Fig. 8).

The entropy change Δ*S* during the R-phase martensitic transition can be evaluated from the specific heat jump Δ*C*_*P*_. First, the Δ*C*_*P*_ value was estimated after subtracting a smooth lattice background (illustrated as a solid line in Fig. 7). This was done by fitting the *C*_*P*_(*T*) data far from the transition region^27^. The entropy change for the Ti_50_Ni_50-*x*_Fe_*x*_ SMAs with *x* = 2.0–6.0 was then evaluated by integrating Δ*C*_*P*_/*T* across the transition (see the inset (a) of Fig. 8), and the deduced Δ*S* values are listed in Table 1. From this estimation, it is found that the Δ*S* value during the R-phase transition decreases gradually with increasing Fe content above *x* = 3.0^19,26,27^. Likewise, the enthalpy change, Δ*H*, across the R-phase transition for these SMAs also decreases with increasing *x* (Table 1). The Δ*H* values of these SMAs were obtained by using the differential scanning calorimetry data^32^. These findings can be attributed to the lowering of the enthalpy difference between the martensite and austenite phases with the change in composition, as well as the stabilization of the B2 structure^24^. Markedly, the transition temperature (*M*_*s*_) of the R-phase SMAs (*x* = 4.0–6.0) decreases linearly with decreasing Δ*H* (the inset (b) of Fig. 8, see left vertical axis). Similarly, the width of thermal hysteresis Δ*T*_*H*_ of these SMAs also decreases linearly with Δ*H* (the inset (b) of Fig. 8, see right vertical axis). These observations are likely owing to the austenite and martensite phases getting more and more similar with increasing Fe content in these R-phase SMAs, as the R-martensite will be formed with a lower nucleation barrier energy^24^. Accordingly, a smaller width of thermal hysteresis (less than 1 K) is observed for the Ti_50_Ni_44_Fe_6_ due to a lower driving force is required for the formation of R-martensite in this SMA.

## Discussion

Our present study confirms that the introduction of point defects (Fe) into TiNi SMA leads to the stabilization (destabilization) of the B2 (B19′) phase^13^. As a result, a decrease in the transition temperature was observed for the Ti_50_Ni_50-*x*_Fe_*x*_ (*x* ≥ 2) SMAs when compared to parent TiNi (*M*_*s*_ ∼ 295 K). This is essentially due to the fluctuations of concentration associated with the induced point defects^13^. In other words, the Fe atoms destabilize the martensite B19′ phase, which ultimately reduces the transition temperature (see Table 1). This is because of the alteration in the global thermodynamic stability of the B19′ phase by Fe substitution^13^. In addition, the Fe atom also affects the local transition temperature of TiNi and thus the transformation width is altered accordingly (Table 1). This is due to the Fe induced divergent stress field at different Ni sites, which affects the local transition temperature via the induced local lattice distortions^13,29^. This means that Fe atoms introduce local strain fields into the B2 matrix that stabilize the R-martensite phase^34–36^. Hence, the examined SMAs including Ti_50_Ni_46_Fe_4_, Ti_50_Ni_44.5_Fe_5.5_, and Ti_50_Ni_44_Fe_6_ overcome local barriers to form the R-martensite^4,27^. These observations can be attributed to two major factors suggested by Frenzel *et al*.^24^: i) the alloying-driven change in geometry (i.e., the changes in bonding as a result of alloying) and ii) the alloying-induced defects that stabilize the B2 phase.

From a microscopic point of view^13^, at low-content defect concentrations of 2.0 ≤ *x* < 6.0, the nanodomains of martensite (R and B19′) are initially formed as a result of randomly distributed point defects. They are then transformed into a long-range strain-ordered state below the transition temperature. At high defect concentrations of *x* > 6.0, the martensitic transformation is completely suppressed and the strain glass (frozen-disordered strain state) features appear instead^20,29^. This is due to the local field effects induced by point defects that create local lattice distortions in the host TiNi lattice. Interestingly, the local field effect promotes the freezing of local strain ordering by preventing the formation of long-range and ordered martensitic twins^13^. In other words, the volume fraction of macro-sized martensite in the TiNi SMA diminishes gradually with increasing Fe content^14^. Hence, a progressive change in the phase transformation and the corresponding physical properties of the TiNi SMA with Fe substitution was observed (see Figs 1–8).

Furthermore, our results show that the entropy change, Δ*S*, during the R-phase transition of the Ti_50_Ni_50-*x*_Fe_*x*_ (*x* = 4.0–6.0) SMAs decreases with increasing *x*. This demonstrates that the transformation heat (proportional to Δ*S*) decreases with lowering transformation strain, according to the equation of Landau free energy (without applied stress)^27,34^: $\Delta S=-A{e}_{M}^{2}$. Here, *A* is the proportionality constant, *e*_*M*_ is the transformation strain (or lattice distortion) at the transition temperature, and the minus sign indicates that the martensitic transformation always leads to a reduction in entropy. Generally, the induced point defects alter the local lattice structure of the TiNi SMA via induced local strain fields^27,35^. Thus, a noticeable entropy change of about Δ*S* < 1.4 J/mole K across the R-phase transition was observed for the Ti_50_Ni_50-*x*_Fe_*x*_ SMAs with *x* = 4.0–6.0 (see Table 1)^19,27^. However, their value is much smaller than that of the B2 → B19′ transition in the Ti_50_Ni_50_ and Ti_50_Ni_48.5_Fe_1.5_ SMAs (>4.0 J/mole K)^31^. This is consistent with the observations in the literature^4,27^. Our present findings confirm that the Fe concentration has a major impact on the characteristics of the martensitic transformation in these Fe-substituted TiNi SMAs, and the observed features are analogous to those studies on the Ni-, Cu-, and Cr-substituted TiNi SMAs^24–26^.

Moreover, it is noticed that the *κ*_*L*_(*T*) of the Ti_50_Ni_50-*x*_Fe_*x*_ SMAs with *x* = 2.0–6.0 do not follow the specific heat *C*_*P*_(*T*) behavior (the peak-shaped anomaly) across the martensitic transformation (see Figs 6 and 7). This means that the Fe-substituted TiNi SMAs do not obey the classical kinetic theory of lattice thermal conductivity (*κ*_*L*_ = *C*_*L*_ *νl*) near the martensitic transition^27,37^. Here, *C*_*L*_,*ν*, and *l* are the phonon specific heat, phonon drift velocity, and mean free path, respectively. Such an observation contradicts to the results found in the Ti_50_Ni_50_ and Ti_50_Ni_48.5_Fe_1.5_ SMAs that both samples show the peak-shaped anomalies in the *C*_*P*_(*T*) and *κ*_*L*_(*T*) across the B2 → B19′ transition^31^. This is most likely due to the absence of soft phonon modes in the Ti_50_Ni_50-*x*_Fe_*x*_ system^27,31^. Such a finding is similar to the observations made in our recent work on the R-phase TiNi-based SMAs^27^. In conclusion, the Fe substitution induces a change in the crystal structure of the TiNi SMA which results in a gradual lessening of the difference between martensite and austenite lattices and hence the decrease in width of thermal hysteresis with increasing *x* is observed. In addition, the stabilization of B2 phase by Fe substitution is attributed to the variation in the electronic structure, which ultimately leads to the reduction in martensitic start temperature *M*_*s*_. Overall, the significant changes in martensitic transformation characteristics have emerged for TiNi SMA after Fe substitution. That is, the substituent concentration has a significant effect on the martensitic start temperature (*M*_*s*_) and transformation width (Δ*T*) of the Ti_50_Ni_50-*x*_Fe_*x*_ SMAs (Table 1), especially for the compounds with *x* > 3.0.

## Summary

The temperature-dependent thermal and transport properties of the Ti_50_Ni_50-*x*_Fe_*x*_ (*x* = 2.0–10.0) SMAs were investigated by means of electrical resistivity, the Seebeck coefficient, thermal conductivity, and specific heat measurements. Our study revealed that the Ti_50_Ni_48_Fe_2_ and Ti_50_Ni_47_Fe_3_ SMAs undergo a two-step martensitic transition (B2 → R and R → B19′), which is distinct from a transition (B2 → B19′) that occurs in the parent TiNi. With further Fe substitution (4.0 ≤ *x* ≤ 6.0), a one-step B2 → R transition was observed for the Ti_50_Ni_46_Fe_4_, Ti_50_Ni_44.5_Fe_5.5_, and Ti_50_Ni_44_Fe_6_ SMAs, accompanied with a complete destabilization of the B19′ phase. The strain glass characteristics were seen for the alloys Ti_50_Ni_42_Fe_8_ and Ti_50_Ni_40_Fe_10_. These findings are essentially attributed to the induced change in the local lattice structure by Fe point defects. Most importantly, we decisively establish that Ti_50_Ni_44_Fe_6_ is the boundary composition SMA, which divides the martensite (*x* ≤ 6.0) and strain glass (*x* > 6.0) state compounds of the Ti_50_Ni_50-*x*_Fe_*x*_ SMAs. This conclusion was validated by a careful comparison of the transition characteristics and physical properties between Ti_50_Ni_44_Fe_6_ and Ti_49_Ni_45_Fe_6_ SMAs. The variation in the characteristics of martensitic transformation in the TiNi SMA with Fe substitution (Ti_50_Ni_50-*x*_Fe_*x*_) can be attributed to the induced local lattice deformations. This primarily resulted from the induced local strain fields by the Fe point defects. Most importantly, the transformation characteristics, such as martensitic start temperature (*M*_*s*_) and width of thermal hysteresis (Δ*T*_*H*_) decrease noticeably with increasing Fe content (*x*). Similarly, during the martensitic transition, both entropy change (Δ*S*) and enthalpy change (Δ*H*) were also found to decrease with Fe content. Remarkably, the *M*_*s*_ and Δ*T*_*H*_ values of the R-phase Ti_50_Ni_50-*x*_Fe_*x*_ SMAs (4.0 ≤ *x* ≤ 6.0) decrease linearly with decreasing Δ*H*. Overall, the substituent concentration has a significant influence on the martensitic transformation characteristics of the Ti_50_Ni_50-*x*_Fe_*x*_ SMAs, leading to a continuous evolution of phase transformation in the Fe-substituted TiNi SMAs.

## Methods

Samples of Ti_50_Ni_50-*x*_Fe_*x*_ (*x* = 2.0–10.0 at.%) SMAs were fabricated using a vacuum arc re-melter, which described elsewhere^32,33^. Briefly, high-purity raw materials consisting of titanium (4 N), nickel (4 N), and iron (3 N) were melted six times using the re-melter to form ingots of the Ti_50_Ni_50-*x*_Fe_*x*_ samples. Here, the weight loss for each ingot is less than 1 × 10^−4^. The ingots obtained were then hot-rolled individually at 1173 K into a plate with a thickness of about 2 mm using a commercial rolling machine (DBR150 × 200 2HI-MILL, Daito Seiki Co, Japan). Subsequently, the sample plates were solution heat-treated at 1173 K for 1 h, followed by water quenching to cool the samples to room temperature (RT). Finally, the surface oxide layer of the samples was removed using an etching solution of HF:HNO_3_:H_2_O (1:5:20 volumes). In addition, the Ni-rich Ti_49_Ni_45_Fe_6_ sample was prepared to explore the influence of excess Ni on the boundary composition (*x* ≤ 6.0) of the Ti_50_Ni_50-*x*_Fe_*x*_ SMAs^19,20^.

For transport and thermal measurements, the sample plates were cut into a rectangular parallelepiped shape with dimensions of about 1.5 × 1.5 × 5.0 mm^3^ using a low-speed diamond cutter. The temperature-dependent electrical resistivity of the Ti_50_Ni_50-*x*_Fe_*x*_ samples was measured using a standard four-probe method. The Seebeck coefficient and thermal conductivity measurements on the TiNi-based SMAs were carried out simultaneously in a closed-cycle refrigerator using a direct heat pulse technique. The specific heat data for these SMAs were obtained using a high-resolution ac calorimeter with chopped light as a heat source. More details about these measurement techniques can be obtained elsewhere^25–28,38^. The presented electrical and thermal measurement systems are all equipped with a calibrated silicon diode thermometer (Lake Shore model DT-470-SD) and all data presented in the manuscript were recorded with a slow heating rate of about 20 K/h and reproducibility better than 2%. The experimental errors in the temperature measurement, according to the manufactory specifications, are ±0.25 K from 2 to 100 K and ±0.50 K from 100 to 300 K.
